# Mr. Goodhall, on Vaccination

**Published:** 1808-10

**Authors:** J. Goodhall

**Affiliations:** Epworth


### *To the Editors of the Medical and Physical Journal.*

GENTLEMEN,

ON June 28, 1807, I inoculated a child four months old for the cow-pox; the pustule on the arm rose well, and was at the height about the tenth day, but I in general prefer taking matter on the eighth or ninth day, which I did on the latter day in this case. Mrs. E. mother to the child, informed me there was a spot, as she termed it, upon the inside of one thigh, and on examining it, I told her I thought it was the cow-pox, but it was about three days later than the inoculated one; I requested she would be

---

be kind enough to take care it was not rubbed off, and I would call again in two or three days, and look at it; when I was agreeably surprised to see the opinion I entertained of it, verified; it was in every respect, a well defined pustule. I inoculated two children with matter taken from it, which had the desired effect on them both; the pustules were at the height on the 9th day, and I continued to inoculate with matter from that source, some time afterwards, without any deviation from the genuine vaccine pock. It is a well known fact, some children are often very uncomfortable for many hours, for want of cloths being more frequently applied; and in consequence of such neglect, their thighs are in such a state of irritation, that they will, if possible, get their hands to the *part* and rub or scratch them, I therefore thought the infection might have been communicated in that way; but Mrs. E. at that time had the whole management of her child, and on particular inquiry, I was informed he never attempted to put his hands either to the arm or thigh, so that the infection could not be communicated by any artificial means, but must have entered the system by the power of absorption. I have read a great deal in the Medical Journal, the Edinbugh Surgical Journal, and other publications, respecting the vaccine disease, but don't recollect a similar instance, related by any of your correspondents; and thinking there was something novel in this case, I have taken the liberty to send you the particulars, which if you think merits your attention, you are requested to publish it in your much esteemed Journal, in such way as you think proper, with my best wishes for whatever promotes the improvement of Medical Science in general, but the cow-pox in particular.

I am, &c.

J. GOODHALL,

*Epworth, Sept.* 8, 1808.

